# Liver Injury Secondary to Anti-TNF-Alpha Therapy in Inflammatory Bowel Disease: A Case Series and Review of the Literature

**DOI:** 10.1155/2014/956463

**Published:** 2014-01-19

**Authors:** Ravish Parekh, Nirmal Kaur

**Affiliations:** Inflammatory Bowel Disease Center, Henry Ford Health System, 39450 West Twelve Mile Roud, Novi, MI 48377, USA

## Abstract

*Background*. Biologic therapy to inhibit tumor necrosis factor-alpha (TNF-**α**) is an effective, safe treatment for patients with inflammatory bowel disease (IBD). All TNF-**α** inhibitors have been associated with liver toxicity, but many of these cases have been reported in patients receiving therapy for rheumatologic disease. Herein we report the first single-center case series of TNF-**α** antagonist related liver injury in patients with IBD. *Methods*. A retrospective case series was performed at the Henry Ford Inflammatory Bowel Diseases Center. IRB approval was obtained. *Results*. 2 patients were treated with infliximab, whereas the 3rd patient was treated with adalimumab for IBD. All 3 patients had negative viral markers, normal autoimmune serologies, and normal biliary imaging studies. Liver biopsy was performed in all 3 patients, and evidence of portal inflammation was seen. Liver enzymes normalized after discontinuation of therapy in all patients, and no long term effects have been observed. One patient was successfully transitioned from infliximab to adalimumab without relapse of either IBD or liver injury. *Conclusion*. Liver injury secondary to TNF-**α** antagonist is an underrecognized, important clinical entity with potentially serious consequences. The mechanism of drug-induced injury is idiosyncratic. Larger cohort studies are needed to establish risk factors and injury patterns related to hepatotoxicity in these patients.

## 1. Introduction

Biologic therapy to inhibit tumor necrosis factor-alpha (TNF-*α*), a proinflammatory cytokine, has become a widely used, safe, and effective treatment for patients with inflammatory bowel disease (IBD) [1]. Since 2008, the number of patients treated with TNF-*α* antagonists has rapidly increased, and as of 2011 more than 1,500,000 people have been exposed to infliximab therapy [2]. However, the side effect profile of these medications is still being established. TNF-*α* antagonists have been associated with the reactivation of latent tuberculosis, reactivation of hepatitis B, demyelinating neurologic disease, and congestive heart failure, among other adverse effects [3]. Cases of TNF-*α* antagonist-induced liver toxicity have been reported, but mainly in patients receiving therapy for rheumatologic disease [4–8]. Little has been reported about anti-TNF-*α* related hepatotoxicity in patients with IBD [2, 4, 9–12]. Clinician awareness of the adverse effects of commonly used therapies is paramount for safe administration of treatment. Herein we report the first single-center case series of anti-TNF-*α* related liver injury in patients with IBD.

## 2. Methods and Materials

The Henry Ford Health System Institutional Review Board approved this retrospective case series. All patients were seen at the Henry Ford Health System Inflammatory Bowel Disease Center in Novi, Michigan. Patient charts were reviewed and clinical information was extracted.

## 3. Ethical Considerations

Since this study required only chart review and no patient intervention, ethical considerations were minimal. Patient privacy was maintained with de-identification of all study documents.

## 4. Results

All results are listed in Table 1.

### 4.1. Subject 1

A 49-year-old female with a 15-year history of ulcerative pancolitis was initially treated with 5-aminosalicylic acid (5-ASA) derivatives, with good control for 12 years. Due to an increase in symptoms as well as moderately severe colitis seen on endoscopy, infliximab was added at 5 milligrams per kilogram (mg/kg) at weeks 0, 2, 6, and then every 8 weeks. After 6 months, due to persistent symptoms as well as persistent endoscopic disease severity, the infliximab dose was escalated to 10 mg/kg every 8 weeks. Prior to infliximab initiation, liver function tests were normal. After 18 months of infliximab therapy, liver enzymes were found to be elevated on routine serologic check: alanine aminotransferase (ALT) elevated to 85; aspartame aminotransferase (AST) was 104, alkaline phosphatase (ALP) elevated to 518, and bilirubin remained normal. The patient remained asymptomatic, with no features of compromised hepatic function, both subjectively and objectively. Autoimmune liver panel, including antinuclear antibody (ANA), antismooth muscle antibody (ASMA), and anti-liver-kidney-mitochondrial antibody (anti-LKM), was negative. Serologic markers for Hepatitis A, B, and C were also negative, including Hepatitis A Immunoglobulin M (IgM), Hepatitis B surface antigen (HBsAg), Hepatitis B core antibody (cAb) IgM, Hepatitis C antibody (HCV Ab), and Hepatitis C RNA (HCV RNA). The patient underwent magnetic resonance cholangiopancreatography (MRCP), which showed no evidence of primary sclerosing cholangitis. She ultimately underwent a liver biopsy, which showed minimal chronic portal inflammation with lack of any autoimmune features or fibrosis. After three months of persistent liver enzyme abnormalities, infliximab was discontinued. Twenty weeks after discontinuation of infliximab, ALT normalized to 21 and AST trended down to 37 and ALP to 142. Despite our discussion of adalimumab therapy or colectomy, the patient elected to avoid all medication except oral mesalamine. She has remained minimally symptomatic, with minimal mucosal activity on follow-up colonoscopy at 6 months.

### 4.2. Subject 2

A 34-year-old female with a 9-year history of mild ulcerative pancolitis developed worsening disease both symptomatically as well as endoscopically. Therapy with infliximab 5 mg/kg, at standard induction dosing and maintenance every 8 weeks, was prescribed, with good symptomatic response. Prior to initiation of infliximab, liver enzymes were within the normal range. After 2 infusions, liver enzymes elevated: ALT was 173 and AST was 160. Following the 3rd infusion at week 6, the patient's liver enzymes elevated even further: ALT was 300 and AST was 259. Autoimmune serologies including ANA, ASMA, and anti-LKM antibody and viral hepatitis panel including hepatitis A IgM, HBsAg, HBcAb IgM, HCV Ab, and HCV RNA were all negative. Alpha-1-antitrypsin levels were also normal. MRCP was unrevealing. Liver biopsy demonstrated chronic periportal inflammation with presence of eosinophils and focal stage 1 fibrosis. No autoimmune features were seen. Given the extent and persistence of enzyme elevation, infliximab was discontinued after 3 infusions. Liver enzymes improved after discontinuation of infliximab. The patient was started on adalimumab therapy after 8 weeks of discontinuation of infliximab. Liver enzymes continued to improve even after discontinuation of infliximab and subsequent initiation of adalimumab. After 6 months of treatment with adalimumab, with concomitant ASA therapy, this patient has continued to be in clinical and endoscopic remission, with liver enzymes being in the normal range.

### 4.3. Subject 3

**Figure 1:**
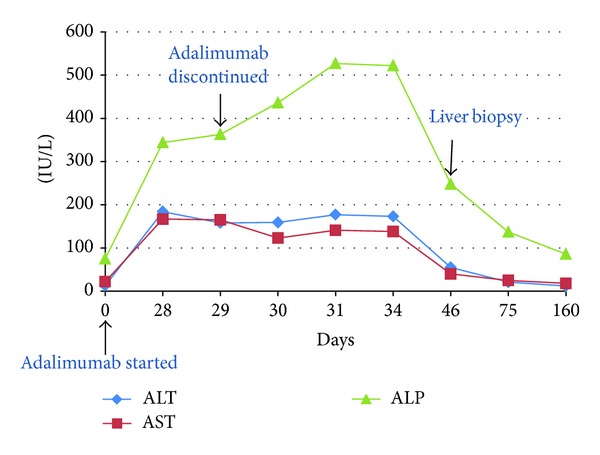
Graphic summary of biochemical liver injury with administration of adalimumab in Subject 3. Aspartate aminotransferase (AST), alanine aminotransferase (ALT), and alkaline phosphatase (ALP).

**Figure 2:**
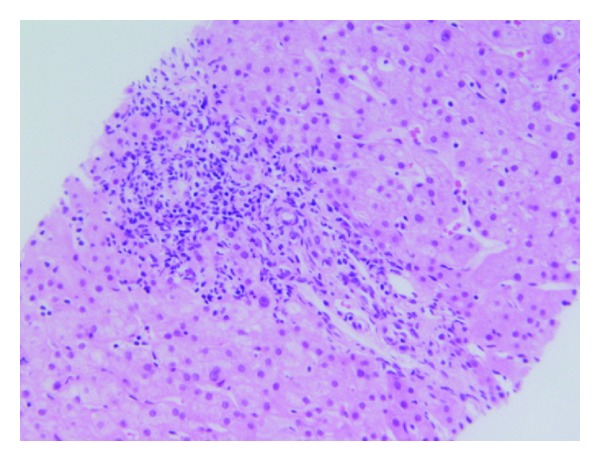
Liver biopsy showing portal inflammation in Subject 3 (hematoxylin and eosin stain, magnification ×200).

**Figure 3:**
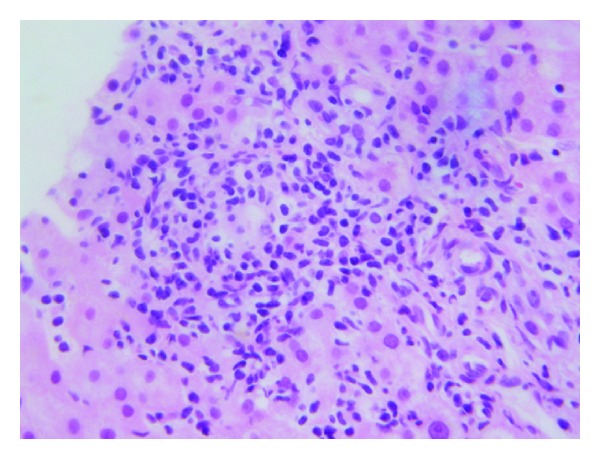
Liver biopsy showing lymphocytic and plasma cell infiltrate in Subject 3 (hematoxylin and eosin stain, magnification ×400).

A 19-year-old female with a 7-year history of severe, fistulizing, ileocolonic, and perianal Crohn's disease was initially treated with ASA, azathioprine, and infliximab. Her disease was refractory to medical management and she underwent total colectomy and permanent end-ileostomy. Given the patient's young age of disease onset and severity of disease, therapy with adalimumab at standard induction dosing (160 mg at week 0, 80 mg at week 2, and 40 mg every other week thereafter) was initiated three months postoperatively to prevent postoperative recurrence of Crohn's disease [13]. Liver enzymes prior to initiation of adalimumab were normal. Four weeks after drug initiation, the patient was hospitalized with complaints of nausea, abdominal pain, and diarrhea. She was found to have elevated liver enzymes, with ALT 184 and AST 167 (Figure 1). Viral hepatitis panel including Hepatitis A Ab IgM, HBsAg, HBcAb IgM, HCV Ab, and HCV RNA was negative. Autoimmune workup with ANA, ASMA, AMA, and Anti LKM Ab was negative as well. MRCP showed an unremarkable biliary system. The patient underwent liver biopsy, which showed lobular and chronic portal inflammation with microgranulomas. The infiltrate included lymphocytes and plasma cells with rare eosinophils and neutrophils. Also, histologically, occasional lymphocytic infiltrates of the bile duct epithelium were seen, focally resembling a florid ductal lesion (Figures 2 and 3). Given the temporal relationship of liver injury to the initiation of adalimumab, as well as the degree of symptomatic involvement, adalimumab therapy was discontinued. After six weeks of drug discontinuation, liver enzymes normalized (Figure 1). This patient has not initiated any further therapy. At 6 months of follow-up, her disease has remained quiescent symptomatically. A discussion regarding azathioprine initiation with close liver monitoring is planned for her follow-up visit.

## 5. Discussion

Our study reports the largest, single-center case series of anti-TNF-*α* drug-induced liver injury in patients with IBD. A recent case series by Ghabril and colleagues [2] reported 6 cases of anti-TNF-*α* drug-induced liver injury from United States Drug Induced Liver Injury Network database, over 8 years, between 2003 and 2011. Our experience of three such cases, seen in one region, over the course of one year, suggests that the incidence of this adverse effect may be more prevalent than is currently recognized. Furthermore, because biologic agents have been utilized for rheumatologic conditions with much more frequency than that of IBD, much of the safety data pertaining to biologic therapy is published in the rheumatology literature. Clinician awareness of this adverse effect is likely lower amongst gastroenterologists.

Liver injury secondary to TNF-*α* antagonist can present with either a hepatocellular pattern or an autoimmune pattern. Several reported cases of TNF-*α* antagonist related liver injury describe a predominantly hepatocellular pattern [2, 4, 5, 10, 14, 15]. Autoimmune injury with positive autoantibodies, including ANA, ASMA, and Anti-LKM antibody, along with classic histologic features of autoimmune hepatitis such as interface hepatitis, lymphoplasmacytic infiltrate, and bridging fibrosis has been reported as well [7–9, 12, 16–19]. In the case series reported by Ghabril and colleagues, patients with autoimmune features experienced longer latency times and higher peak ALT levels compared to those lacking autoimmune features [2]. Cholestatic hepatitis secondary to anti-TNF-*α* agents has been reported [2, 6, 10, 20] and in one case report, hepatic necrosis resulted in fulminant liver failure requiring urgent liver transplantation [11]. There has also been a case report of hepatocellular carcinoma in a patient treated with a TNF-*α* antagonist in combination with azathioprine [21]. In our series, all three patients experienced hepatocellular injury both biochemically as well as histologically, with negative autoimmune serologies. Our patients did exhibit microscopic features of hepatitis, without interface hepatitis or lymphoplasmacytic infiltration. This pattern of injury is more consistent with drug-induced injury rather than acute autoimmune hepatitis, as evidenced by histology as well as resolution after discontinuation of the offending agent. The hepatitis was not fatal and no serious injury was reported.

We initially postulated that the variation in injury pattern could be secondary to variables such as concomitant medications or dosage of medications. However, review of our cases and the existing literature showed no such relationship. Dosage of TNF-*α* antagonists did not correlate with liver injury in our case series. Amongst our patients, Subject 1 received therapy with high dose infliximab (10 mg/kg every 8 weeks) when hepatotoxicity was documented. However, Subjects 2 and 3 received standard doses of infliximab (5 mg/kg every 8 weeks) and standard induction dosing of adalimumab (160 mg at week 0, 80 mg at week 2, and 40 mg every other week thereafter), respectively, when liver injury was noticed. Review of previously reported cases revealed that the onset of hepatitis has been noticed with single administration of infliximab in one case [10], but after prolonged administration of the medication in other cases.

Latency time to the development of liver toxicity was also variable. In our case series, Subject 1 developed liver toxicity after 18 months of infliximab, whereas toxicity developed within 3 months in Subjects 2 and 3. Hepatotoxicity was not related to any particular TNF-*α* antagonist, and patient age also varied among our three patients. The variability of histology, dosage, time to toxicity, and presence of concomitant medications, in our patients as well as in review of published cases, highlights the idiosyncratic nature of this drug-induced liver injury. As clinician awareness of this entity increases, and more cases are detected, hopefully distinct patterns of injury will be delineated so that early detection can take place and fulminant liver failure can be prevented [17, 22, 23].

The lack of cross-reactivity between the TNF-*α*-antagonist-related liver injury, as seen in multiple case reports [2, 4, 14, 15, 18, 19, 24, 25], is likely due to the difference in molecular structure of these agents. Since infliximab is a chimeric IgG1 monoclonal antibody and adalimumab is a fully humanized IgG1 monoclonal antibody and etanercept is a fusion of recombinant soluble TNF-a receptor type 2 with an Fc domain of human IgG1, antibody formation and immune-mediated reactions are more likely to develop against infliximab rather than the latter two molecules. This phenomenon is supported by the above-referenced case studies, in which patients with infliximab-induced hepatitis have successfully transitioned to therapy with etanercept and adalimumab. In our case series, Subject 2 experienced infliximab-induced hepatocellular injury, which resolved after discontinuation of the drug. Therapy was successfully transitioned to adalimumab with no evidence of hepatocellular injury on follow-up laboratory testing, and the patient's disease remained in clinical and endoscopic remission. Thus, in the setting of infliximab-related liver injury, transitioning to an alternate TNF-*α* antagonist is a reasonable therapeutic strategy.

One clear limitation of our study is the lack of formal causality assessments. However, given the thorough clinical evaluation pursued in each of the three patients, the strong temporal relationship between the drug and liver injury, the lack of other causal factors such as acetaminophen, alcohol, nonalcoholic fatty liver disease, or metabolic syndrome, and, most importantly, the resolution of injury after discontinuation of the offending agent, the authors determined that suspicion for drug-induced injury was sufficient. A second limitation of the study is the small number of patients. However, given the retrospective, observational nature of this study, we believe that our case series is indicative of a larger, underrecognized clinical entity.

In conclusion, TNF-*α* antagonist-related liver injury is an underrecognized, important clinical entity with potentially serious consequences, such as liver failure. The mechanism of drug-induced injury can be either hepatocellular or autoimmune, and injury can occur irrespective of dose, number of infusions or injections, or time. Given the increasing utilization of TNF-*α* antagonists, many more patients will be at risk for hepatotoxicity in the future. Larger cohort studies are needed to establish the risk factors and injury patterns related to hepatotoxicity in these patients. Currently, most IBD clinics do not utilize protocols to monitor liver function in patients receiving therapy with TNF-*α* antagonists, but perhaps this practice should be considered.

## Figures and Tables

**Table 1:** 

Patient	Patient 1	Patient 2	Patient 3
Age	49	34	19

Sex	Female	Female	Female

Disease	UC	UC	Crohn's

Concomitant drugs	Mesalamine, synthroid	Mesalamine, synthroid, sertraline	None

TNF-*α*-antagonist	Infliximab	Infliximab	Adalimumab

Time to liver toxicity	18 months	3 months	1 month

Peak ALT	104	300	184

Peak AST	85	259	167

Total bilirubin	0.4	0.4	1.7

Direct bilirubin	0.1	0.1	1

Peak ALP	518	77	527

Albumin	3.3	4	3.2

INR	1.07	0.98	1.14

ANA	Negative	Negative	Negative

Anti-LKM	Negative	Negative	Negative

ASMA	N/A	Negative	Negative

Hep A IgM	Negative	Negative	Negative

Hep B sAg	Negative	Negative	Negative

Hep B core IgM	Negative	Negative	Negative

Hep C Ab	Negative	Negative	Negative

MRCP	Normal biliary system	Normal biliary system	Normal biliary system

Liver Biopsy	Chronic portal inflammation	Chronic portal inflammation	Lobular and chronic portal inflammation with microgranulomas

Outcome	Infliximab discontinued. Liver recovery in 8 weeks. In clinical and endoscopic remission on ASA monotherapy	Switched to Adalimumab. Liver recovery in 8 weeks. In clinical and endoscopic remission	Adalimumab discontinued. Liver recovery in 6 weeks. In clinical remission on no therapy

Ulcerative colitis (UC), Crohn's disease (CD), aspartate aminotransferase (AST), alanine aminotransferase (ALT), alkaline phosphatase (ALP), international normalized ratio (INR), antinuclear antibody (ANA), antismooth muscle antibody (ASMA), antiliver-kidney-mitochondrial antibody (anti-LKM), Hepatitis A immunoglobulin M (IgM), Hepatitis B surface antigen (HBsAg), Hepatitis B core antibody (cAb) IgM, Hepatitis C antibody (HCV Ab), and magnetic resonance cholangiopancreatography (MRCP).
